# Dysferlin-deficiency has greater impact on function of slow muscles, compared with fast, in aged BLAJ mice

**DOI:** 10.1371/journal.pone.0214908

**Published:** 2019-04-10

**Authors:** Erin M. Lloyd, Hongyang Xu, Robyn M. Murphy, Miranda D. Grounds, Gavin J. Pinniger

**Affiliations:** 1 School of Human Sciences, the University of Western Australia, Perth, Western Australia, Australia; 2 Department of Biochemistry and Genetics, La Trobe Institute for Molecular Science, La Trobe University, Melbourne, Victoria, Australia; University of Minnesota Medical Center, UNITED STATES

## Abstract

Dysferlinopathies are a form of muscular dystrophy caused by gene mutations resulting in deficiency of the protein dysferlin. Symptoms manifest later in life in a muscle specific manner, although the pathomechanism is not well understood. This study compared the impact of dysferlin-deficiency on *in vivo* and *ex vivo* muscle function, and myofibre type composition in slow (soleus) and fast type (extensor digitorum longus; EDL) muscles using male dysferlin-deficient (dysf^-/-^) BLAJ mice aged 10 months, compared with wild type (WT) C57Bl/6J mice. There was a striking increase in muscle mass of BLAJ soleus (+25%) (p<0.001), with no strain differences in EDL mass, compared with WT. *In vivo* measures of forelimb grip strength and wheel running capacity showed no strain differences. *Ex vivo* measures showed the BLAJ soleus had faster twitch contraction (-21%) and relaxation (-20%) times, and delayed post fatigue recovery (ps<0.05); whereas the BLAJ EDL had a slower relaxation time (+11%) and higher maximum rate of force production (+25%) (ps<0.05). Similar proportions of MHC isoforms were evident in the soleus muscles of both strains (ps>0.05); however, for the BLAJ EDL, there was an increased proportion of type IIx MHC isoform (+5.5%) and decreased type IIb isoform (-5.5%) (ps<0.01). This identification of novel differences in the impact of dysferlin-deficiency on slow and fast twitch muscles emphasises the importance of evaluating myofibre type specific effects to provide crucial insight into the mechanisms responsible for loss of function in dysferlinopathies; this is critical for the development of targeted future clinical therapies.

## Introduction

Dysferlinopathies are a clinically heterogeneous group of muscle disorders that arise from mutations in the dysferlin gene (*DYSF*) that reduce expression of functional dysferlin protein [1]. Clinically, dysferlinopathies are described as limb-girdle muscular dystrophy type 2B or Miyoshi myopathy, with initial weakness in the proximal limb girdle or distal limb muscles respectively [2], although gradations across phenotypes are now more widely recognised [3]. Most patients with dysferlinopathy become wheelchair bound within 10–20 years after diagnosis, and currently there is no treatment.

Dysferlin is a member of the ferlin family of transmembrane proteins, which have roles in protein vesicle fusion and trafficking [4]. Dysferlin is present in many cell types including macrophages, adipocytes, smooth muscle and vascular endothelial cells [5], but it is highly expressed in skeletal muscle. The localisation of dysferlin to the sarcolemma and transverse-tubules (T-tubules) in skeletal muscle [6, 7], suggests a possible role in Ca^2+^ handling associated with excitation-contraction (EC) coupling [8]. Dysferlin is associated with normal developing T-tubules, and abnormally configured T-tubules are found in dysf^-/-^ skeletal muscle [8]. Dysferlin also interacts with Ca^2+^ handling proteins, including calsequestrin-1, ryanodine receptor and the dihydropyridine receptor [6, 9]. However, the extent to which dysferlin-deficiency causes dysfunctional Ca^2+^ handling is not clear, nor are the mechanisms for how this might translate to the specific functional clinical deficits characteristic of human dysferlin-deficient (dysf^-/-^) muscles.

Disease onset in dysferlinopathy typically occurs post growth, in early adulthood. Dysferlinopathy is characterised by progressive skeletal muscle weakness, increased fatigability [10], accumulation of lipid droplets in slow twitch myofibres [5, 11], complement activation [11], muscle wasting characterised by atrophy, autolysis/proteolysis and autophagy [12, 13], inflammation, increased oxidative stress, [14, 15] and, in later stages of the disease, replacement of muscles by fat [1, 5, 11, 16]. Histopathological differences between dysf^-/-^ slow and fast myofibres have been reported, with dysf^-/-^ slow oxidative myofibres containing many lipid droplets, especially in humans [5], whereas, tubular aggregates are present in fast glycolytic myofibres [17](Grounds MD, unpublished observations). The precise molecular mechanisms underlying the dystropathology and how these histopathological features may contribute to the loss of muscle function are not clear [11, 18].

Studies of dysferlin-deficiency typically use dysf^-/-^ mice as they have similar muscle structure to humans and more accurately represent the disease pathology in human dysferlinopathies, compared with dysf^-/-^ zebrafish, roundworm and fruit fly [19, 20]. In general, dysf^-/-^ mice mimic human dysferlinopathies, showing a similar disease progression with late onset of histopathological features [5, 21, 22]. The main dysf^-/-^mouse models, A/J^dysf-/-^ (A/J), SJL/J^dysf-/-^ (SJL/J) and BLA/J^dysf-/-^ (BLAJ; B6.A-Dysf^prmd^/GeneJ), have similar disease manifestation [20] with few changes apparent in young adults aged 3 months, but marked histopathology with replacement of myofibres by adipocytes conspicuous by about 10 months, especially in the psoas and quadriceps muscles [5, 14].

About a dozen published studies have examined muscle function in dysf^-/-^ mice, however these have yielded inconsistent and contrasting results, and often do not replicate the characteristic muscle weakness of human patients with dysferlinopathy (see Table 1). Such inconsistencies between pre-clinical studies may be due partly to the subtle difference in disease severity of the specific dysf^-/-^ mouse strains used [20], and/or the absence of appropriate WT control strains for their comparisons. The age of the mice examined is another important variable; the dystropathology is typically mild in mice aged less than 6 months old, and dysf^-/-^ mice begin to exhibit more marked pathology in some muscles from around 8 months of age [14, 20]. Therefore, it is important to evaluate the functional impact of dysf^-/-^ in older animals, which are more likely to manifest the progressive functional impairment that is expected to result over time from dysferlin-deficiency.

**Table 1:** Summary of prior studies of muscle function in dysferlin-deficient mice.

Dysf^-/-^ strain	Controls	Age (mths)	Sex	Muscles	*In vivo* measures	*Ex vivo* measures	Reference
A/J	A/WySnJ, C57Bl/6J	3	M	Dorsiflexors^b^	Torque at ankle larger in dysf^-/-^		[23]
A/J	A/WySnJ	3–4	M	Dorsiflexors^b^	Torque at ankle no different		[24]
A/J	A/WySnJ, C57Bl/6J	2, 8	M	Soleus EDL		Maximum specific force lower in dysf^-/-^ EDL (2 months)	[25]
A/J	A/JOlaHsd, C57Bl/6J	9	F	Forelimb, Hindlimb, EDL	i. Open field behaviour lower in dysf^-/-^ ii. Forelimb/hindlimb grip strength no different	Maximum specific force no different	[26]
i. A/J ii. BLAJ iii. SJL/J iv. B10.SJL	i. A/WySnJ ii. C57Bl/6J iii. SWR/J iv. C57BL/10J	3–4	M	Dorsiflexors^b^	Torque at ankle lower in dysf^-/-^ (SJL/J, B10.SJLJ)		[21]
A/J, Bl.AJ	C57Bl/6J	3–9	NS^a^	N/A	Open field behaviour lower in dysf^-/-^ (A/J, BLAJ >6 months))		[27]
BLAJ	C57Bl/6J	>20	NS^a^	Forelimb, Hindlimb	i. Open field behaviour lower in dysf^-/-^ ii. Hindlimb grip strength lower in dysf^-/-^		[28]
BLAJ	C57Bl/6J	2–3, 13–15	M/F	N/A	i. Open field behaviour lower in dysf^-/-^ (>3 months) ii. Grip strength (hang test) lower in dysf^-/-^ (>13 months)		[29]
SJL/J	C57BL/6J	2–6	M	Forelimb, Hindlimb, Soleus, EDL	i. Open field behaviour lower in dysf^-/-^ ii. Grip strength higher in dysf^-/-^	Maximum specific force lower in dysf^-/-^ (6 months)	[30]
BL10.SJL-Dysf^jm^/AwaJ	C57Bl/10	3	M	TA (*in situ*)		Maximum specific force no different	[31]
B6.129-Dysf^tm1Kcam^/J	C57Bl/6J	2, 8	NS^a^	EDL	Open field behaviour no different	Maximum specific force no different	[32]
B6.129-Dysf^tm1Kcam^/J	C57Bl/6J	11	NS^a^	Soleus	Gait no different	Fatigue and post fatigue recovery no different	[33]
Dysf^-/-^ 129/SVemst/J	129/SVemst/J	14	NS^a^	N/A	Voluntary wheel running lower in dysf^-/-^		[17]

^a^NS: not specified

^b^ Dorsiflexors muscle group; TA (tibialis anterior), extensor hallucis longus, EDL, and peroneus tertius

While some differences in features of dysf^-/-^ slow and fast myofibres have been reported (mentioned above), we identified only two studies that directly compared muscle function for predominantly slow (soleus) and fast twitch (EDL) muscles [25, 30] with conflicting results. While lower specific force (~92% of WT force) was reported in dysf^-/-^ EDL muscles of A/J male mice aged 2 months, no differences were found at 8 months, nor for the dysf^-/-^ soleus at either age [25]. A similar reduction in maximum force produced *ex vivo* by both dysf^-/-^ EDL and soleus muscles (~85% of control force) was reported in dysf^-/-^ SJL/J male mice aged 6 months [30]; however, this result is complicated by the fact that C57Bl/6J mice were used as the control normal strain for comparison with the dysf^-/-^ SJL/J mice (see Discussion).

These conflicting observations warrant further investigation, due to the important role of impaired muscle function in dysferlinopathies. Therefore, the present study thoroughly investigated the effects of dysferlin-deficiency on a wide range of parameters related to muscle function *ex vivo* for the predominantly slow (soleus) and fast (EDL) twitch skeletal muscles, as well as some measures of whole body function *in vivo*, using mature 10 month old male BLAJ mice where pronounced histopathology is manifested in limb girdle muscles (compared with WT controls).

## Methods

### Ethical approval

All experiments were approved by the Animal Ethics and Experimentation Committee of the University of Western Australia (RA/3/100/1436), in accordance with guidelines of the National Health and Medical Research Council of Australia.

### Animals

This study used 10 month old male C57Bl/6J (the WT parental control strain) and dysf^-/-^ BLAJ mice. Mice were maintained at the Preclinical Animal Facility at the University of Western Australia, housed individually in cages with food and water, maintained in a 12 hour light/dark regime at 20–22°C.

There were two separate experimental groups in this study. **Group 1** involved mice subjected to wheel running (Ex), and consisted of WT + Ex and BLAJ + Ex groups (*n* = 5, 4 respectively) and **Group 2** consisted of WT and BLAJ mice (*n* = 8, 9 respectively) where muscles were sampled, when they reach 10 months old, for *ex vivo* measurements.

### Grip strength measurements

Measures of grip strength and body mass were made on the morning of sampling day for all experimental groups. Forelimb grip strength was measured using a Chatillon Digital Force Gauge (Model DFE-002; AMETEK, FL, USA) following the TREAT-NMD standard protocol “Use of grip strength meter to assess limb strength of mdx mice–DMD_M.2.2.001”. The average of four consecutive grip strength tests was recorded and normalised to body mass (g gBM^-1^).

### In vivo wheel running measurements (Group 1)

Mice were obtained at approximately 8.5 months of age and housed individually in Lafayette Mouse Activity Wheel Chambers (Model 80820; Lafayette Instrument, IN, USA). The apparatus for this experiment in our lab is described previously [34]. The low resistance wheel running capacity of each mouse was measured continuously, and their running patterns were assessed for two weeks. Total distance run (km) and maximum average speed per 15-minute interval (km hr^-1^) for each week was calculated from the raw data. At the end of the experiment, mice (aged 10 months) were euthanised (via intraperitoneal overdose of sodium pentobarbitone) and additional tissues, including soleus, EDL, quadriceps, liver and adipose tissue, were sampled and snap frozen for later analyses in future studies.

### Ex vivo assessment of contractile function for soleus and EDL muscles (Group 2)

Mice were anesthetised using sodium pentobarbitone (40 mg kgBM^-1^) and placed on a heated plate at 37°C to maintain core body temperature. The soleus and EDL muscles were dissected and used as representatives of slow and fast twitch muscles respectively. Mice were then euthanized (by overdose of pentobarbitone) and additional tissues were snap frozen for later analyses. Immediately following dissection, muscles were mounted in an *in vitro* force transducer system (1205A, Aurora Scientific Inc., Aurora, Canada) in an organ bath containing mammalian ringer solution which comprised (in mM): 121 NaCl, 5.4 KCl, 1.2 MgSO_4_.7H_2_O, 25 NaHCO_3_, 5 HEPES, 11.5 glucose and 2.5 CaCl2, bubbled with Carbogen (95% O_2_, 5% CO_2_) and maintained at 25°C. The muscles were stimulated by supramaximal 0.2 ms square wave pulses (701B High Power Bi-phase Current Stimulator, Aurora Scientific Inc., Aurora, Canada). Recording of force and the control of the lever arm was achieved using Dynamic Muscle Control (DMC) software and the data were analysed using Dynamic Muscle Analysis (DMA) software (Aurora Scientific Inc., Aurora, Canada).

At the start of each experiment, the optimal muscle length (L_o_) was identified as the length at which maximal isometric twitch response was produced; the muscles remained at L_o_ for all subsequent measurements. Assessment of contractile function included the twitch characteristics: peak isometric twitch force (P_t_), time to peak twitch force (TTP), half-relaxation time (½RT) and maximum rate of force production (dF/dt), and the force-frequency relationship including maximum tetanic force (P_o_).

Muscle fatigability was assessed using an isometric fatigue protocol. Soleus muscles were stimulated 100 times at 60 Hz for 800 ms once every two seconds (approximately 3.5 minutes); EDL muscles were stimulated 100 times at 70 Hz for 500 ms once every two seconds (approximately 3.5 minutes). Fatigue was measured as the force produced at the end of the stimulation protocol as a percentage of initial force (%P_i_), and post-fatigue recovery was monitored by recording tetanic force (same as fatigue protocol stimulation) at 5 minute intervals for 40 minutes.

Specific force (N cm^-2^) was calculated as force (N) normalised to physiological cross sectional area (CSA, cm^2^). CSA was approximated by the formula: CSA = W/(Lo×FLR×D×100), where W is the wet muscle mass (mg), L_o_ is optimal muscle length (mm), FLR is myofibre/muscle length ratio (EDL = 0.44, Soleus = 0.69)[35], and D is density of muscle (1.06 g mL^-1^)[36].

### Myofibre typing: Myosin heavy chain (MHC) gels

Myofibre myosin heavy chain (MHC) composition was determined by distinguishing four MHC isoforms, MHCIIa, MHCIIx, MHCIIb and MHCI in soleus and EDL muscles from **Group 1**. Muscles were homogenised 20 μg wet weight muscle/μl buffer, which comprised (in mM): 126 K^+^, 36 Na^+^, 1 free Mg^2+^ (10.3 total Mg^2+^), 90 HEPES, 50 EGTA, 8 ATP, 10 creatine phosphate, pH 7.10, pCa (= − log_10_[Ca^2+^])>9, 295±10 mosmol/kg H_2_O. After the homogenisation, muscle samples were denatured by the addition of a 2x denaturing buffer, comprised of 125 mM Tris-HCl, 25% glycerol, 4.6% SDS, 10% mercaptoethanol, 25% sucrose, and 0.001% bromophenol blue. Total protein in samples was separated using SDS-PAGE, adapted for optimal separation of the four MHC isoforms, as previously described [37]. A small amount of each sample was taken and pooled together to create a ‘mixed muscle sample’ (Mixed), which was then loaded onto each gel to generate a calibration curve that demonstrated the relationship between band density and amount of given protein (in arbitrary units). This was used to assign values for each MHC isoform band present in the individual muscle samples. Immediately after running the gels, they were stained with Coomassie brilliant blue G250 (Bio-Rad, Gladesville, NSW, Australia) to visualize the MHC bands. Images were collected, and densitometry of each band performed using Chemidoc MP system and Image Lab version 6.0 (Bio-Rad).

### Analyses

Statistical analyses were performed using IBM SPSS Statistics 24 (IBM Corp., 2012). Data are presented as individual values with horizontal lines indicating mean values ± standard deviation (SD), or single points representing mean ± SD where appropriate. Normality and variance equality were tested using Shapiro-Wilk tests and Levene's Test for Equality of Variances respectively. Data were examined using separate one-way Analyses of Variance (ANOVAs), two-way ANOVAs, or t-tests where appropriate. Post hoc comparisons with Holm-Bonferroni corrections were conducted for each statistical test where appropriate, with statistical significance taken as *p* < 0.05.

## Results

### Body and muscle mass

**Fig 1:**
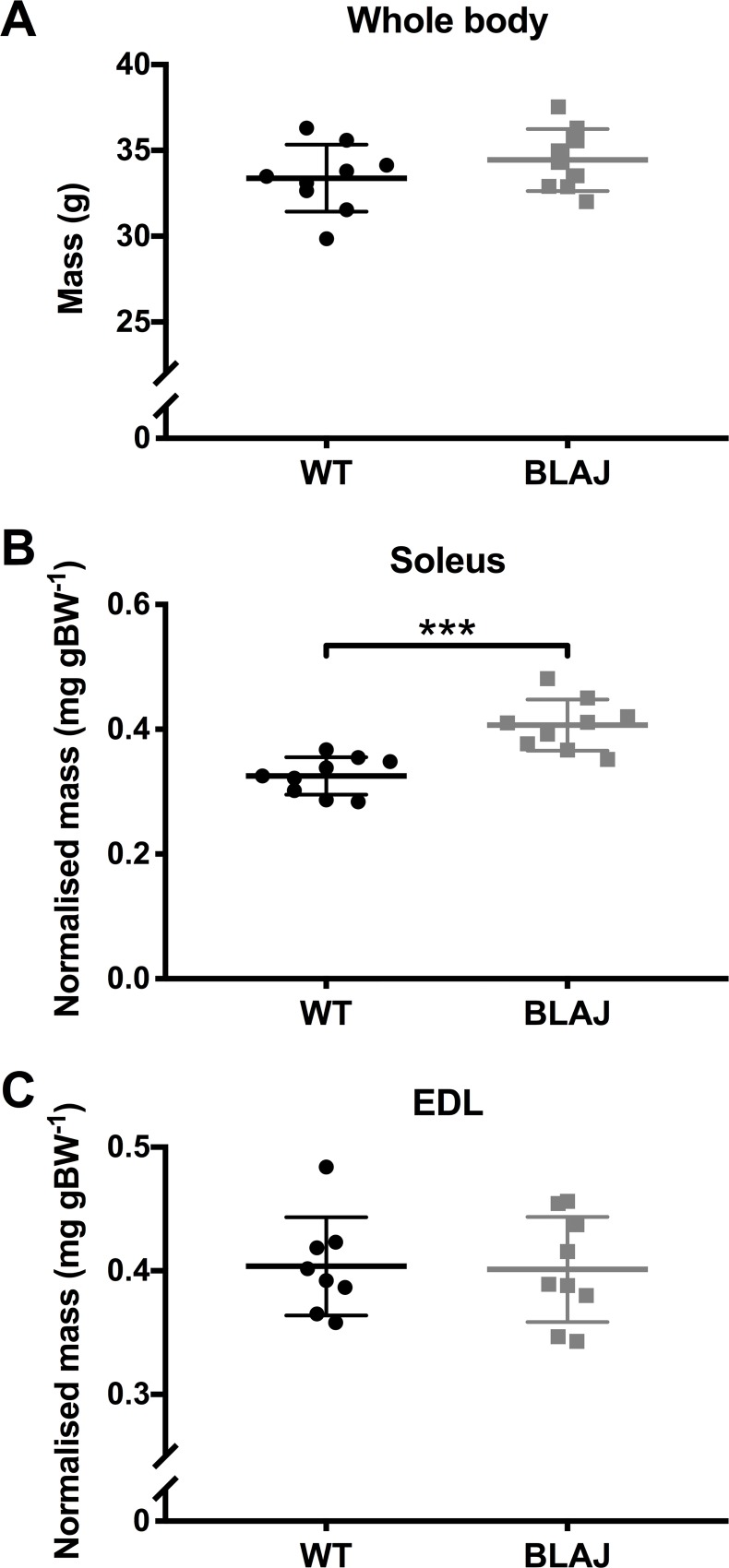
Body and muscle masses of WT and BLAJ mice aged 10 months. (A) Body mass measured at sampling (*n* = 9, 9; WT and BLAJ). (B) Soleus mass normalised to body mass (*n* = 9, 9). (C) EDL mass normalised to body mass (*n* = 8, 9). *** BLAJ significantly different to WT (*p* < 0.0001). Data are presented as individual values with horizontal lines indicating mean ± SD.

Body mass at the time of sampling did not differ between the male WT and BLAJ mice aged 10 months (Fig 1A). The soleus and EDL weights were normalised to body mass. The BLAJ soleus was significantly heavier (~125%) compared with the WT soleus (*p* < 0.001), with no strain difference for the EDL weights (*p*s > 0.05).

### In vivo function measures

**Fig 2:**
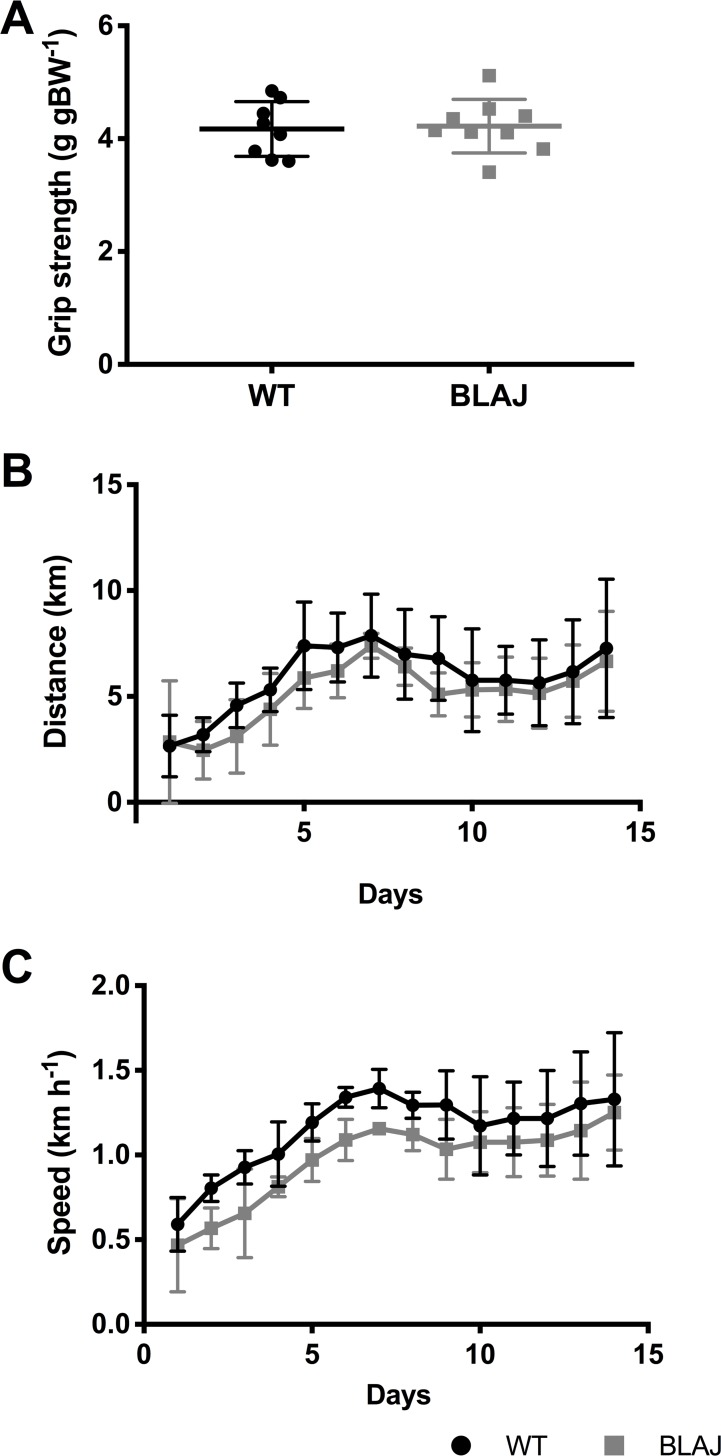
*In vivo* functional measures for WT and BLAJ mice aged 10 months. (A) Grip strength recorded on the day of sampling for WT and BLAJ (*n* = 8, 9). Voluntary wheel running daily total distance run (B) and daily maximum average speed over 15-min interval (C) for WT + Ex and BLAJ + Ex mice aged 10 months (*n* = 5, 4). Data are presented as (A) individual values with horizontal lines indicating mean ± SD or (B, C) mean ± SD.

Forelimb grip strength recorded on the day of sampling (Fig 2A) was not significantly different between strains (*p* > 0.05). Voluntary wheel running measures of daily total distance run and maximum speed (calculated over a 15-min interval) (Fig 2B and 2C) did not differ between WT and BLAJ mice (*p* > 0.05). However, the maximum speed in the BLAJ group tended to be lower than WT over the wheel running regimen (*p* = 0.055, partial η^2^ = 0.43, n = 5, 4 respectively).

### Ex vivo muscle function

**Fig 3:**
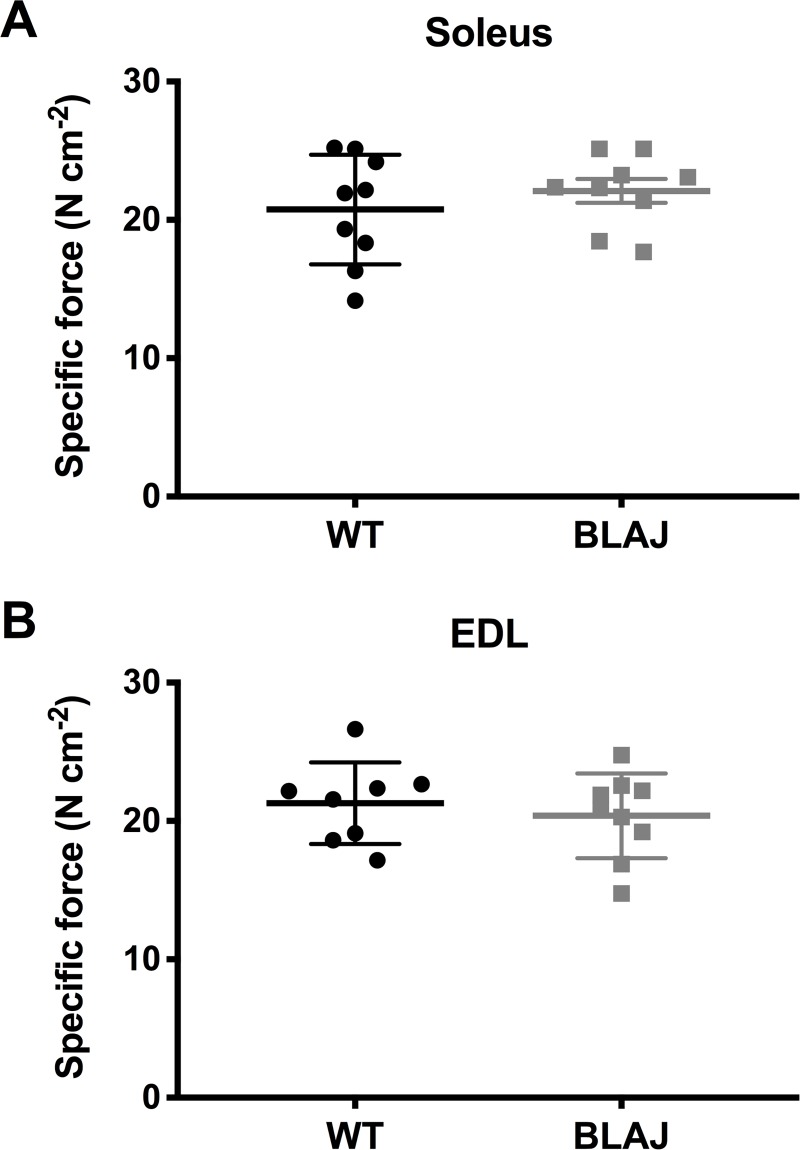
Maximum tetanic force from WT and BLAJ mice aged 10 months. (A) Soleus (*n* = 9, 9) and (B) EDL (*n* = 8, 9). Data are presented as individual values with horizontal lines indicating mean ± SD.

**Fig 4:**
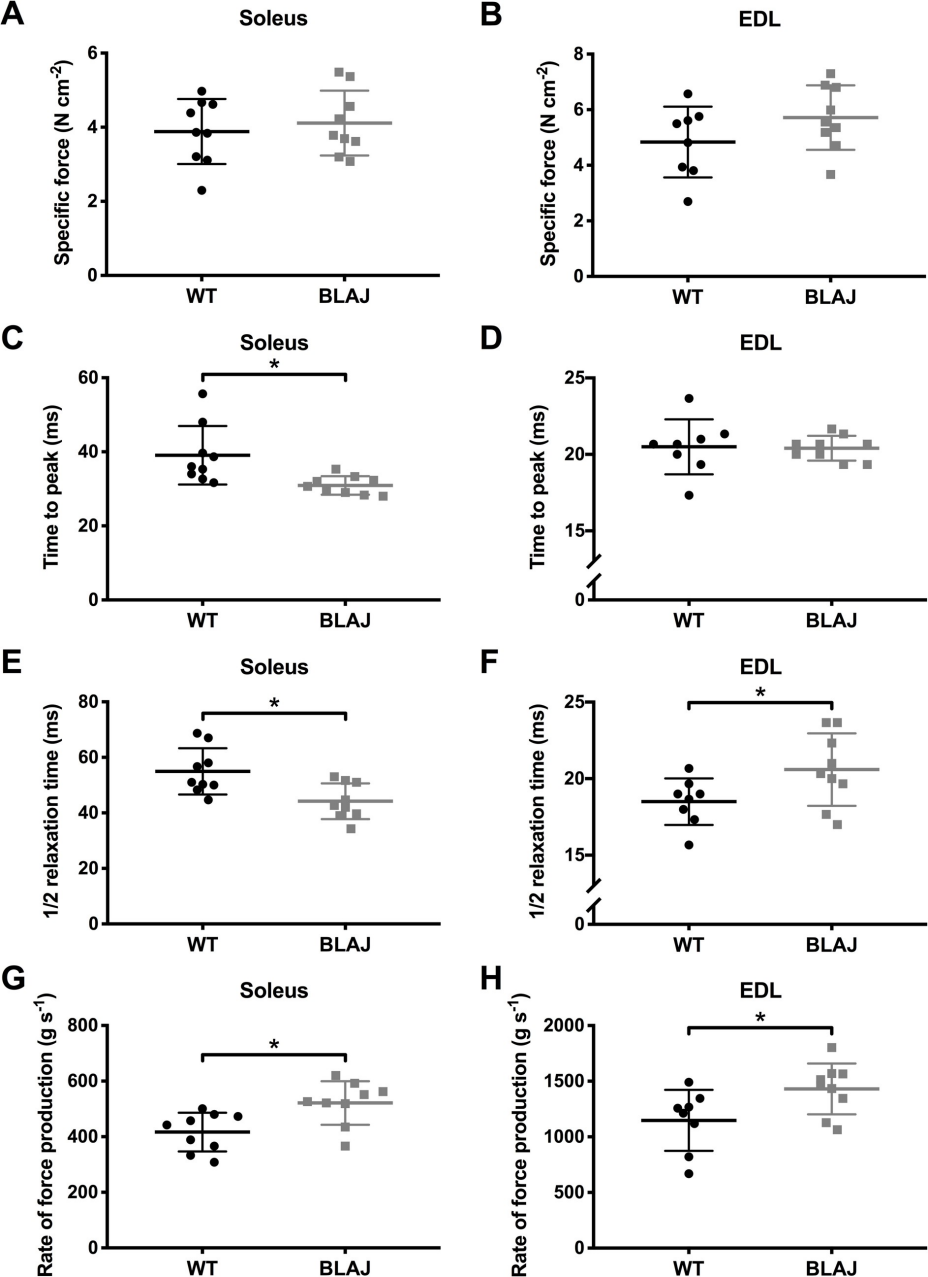
Twitch characteristics from WT and BLAJ mice aged 10 months. Twitch characteristics of the soleus (*n* = 9, 9) and EDL (*n* = 8, 9) consist of peak twitch force (A, B), time to peak twitch force (C, D), 1/2 relaxation time (E, F), and maximum rate of force production (G, H). * BLAJ significantly different to WT (*p*s < 0.05). Data are presented as individual values with horizontal lines indicating mean ± SD.

*Ex vivo* muscle function showed no effect of dysferlin-deficiency on the maximum tetanic force of the soleus or EDL (*p*s > 0.05) (Fig 3). Analysis of the twitch characteristics (Fig 4) of the soleus revealed no differences in peak twitch force between WT and BLAJ (p > 0.05). However, TTP and 1/2RT were significantly shorter in the BLAJ soleus compared with WT (*p* < 0.01 and *p* < 0.05 respectively); these differences are also reflected in the higher dF/dt in the BLAJ soleus (*p* < 0.001). In the EDL, peak twitch force and TTP were unaffected by dysferlin-deficiency (*p*s > 0.05), however, 1/2RT and dF/dt was significantly greater in the BLAJ EDL compared with WT (*p*s < 0.05).

**Fig 5:**
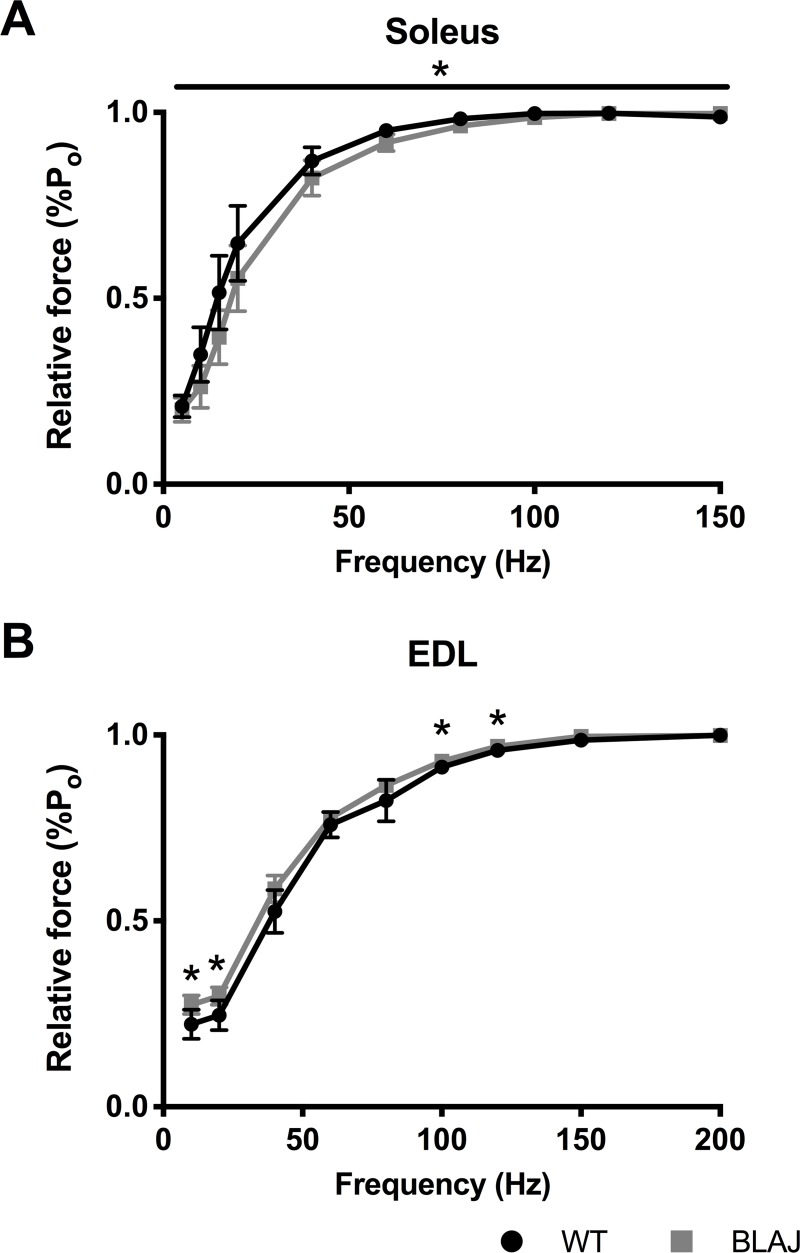
Normalised force-frequency relationship from WT and BLAJ mice aged 10 months. (A) soleus (*n* = 9, 9) and (B) EDL (*n* = 8, 9) muscles. * BLAJ significantly different to WT (*p*s < 0.05). Data are presented as mean ± SD.

The mean of the normalised force frequency curve of the BLAJ soleus was lower than the WT (*p* < 0.05) (Fig 5A), whereas, the force frequency relationship of the BLAJ EDL was higher than WT at 10, 20, 100, and 120Hz (*p*s < 0.05) (Fig 5B).

**Fig 6:**
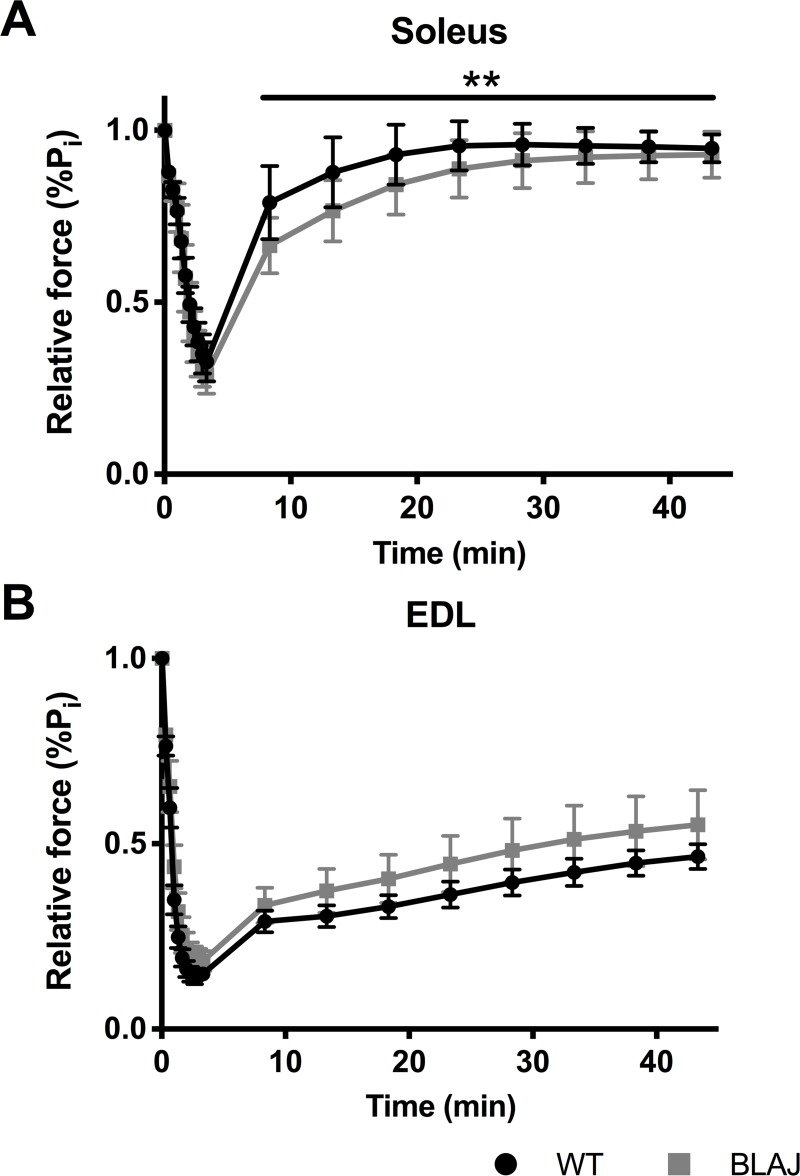
Fatigue and post fatigue recovery from WT and BLAJ mice aged 10 months. (A) soleus (*n* = 9, 9) and (B) EDL (*n* = 7, 9) muscles. The fatigue protocol was approximately 3 minutes, and post fatigue recovery was recorded at 5 minute intervals for 40 minutes after the fatigue protocol. ** significant interaction effect of time and strain post fatigue (*p* < 0.001). Data are presented as mean ± SD.

Dysferlin-deficiency did not impact the susceptibility to fatigue in the soleus and EDL muscles (*p*s > 0.05). The WT and BLAJ soleus muscles fatigued to 32.7% and 28.8% of initial force respectively, whereas the WT and BLAJ EDL muscles fatigued to 14.8% and 18.3% of initial force (Fig 6). After 40 minutes of recovery, soleus force had reached 94.7% and 92.9% of initial force levels for WT and BLAJ mice respectively. However, soleus post fatigue recovery showed a significant interaction effect of time and strain (*p* < 0.001). Pairwise comparisons at each time point did not reach statistical significance, but 5, 10 and 15 minutes post fatigue recovery showed the largest differences (BLAJ lower relative force than WT), suggesting this was likely the source of the overall interaction effect. The post fatigue recovery of the dysf^-/-^ EDL was not significantly different to the WT EDL (*p* > 0.05) reaching 46.5% and 55.1% of initial force levels after 40 minutes.

### Myofibre typing

**Fig 7:**
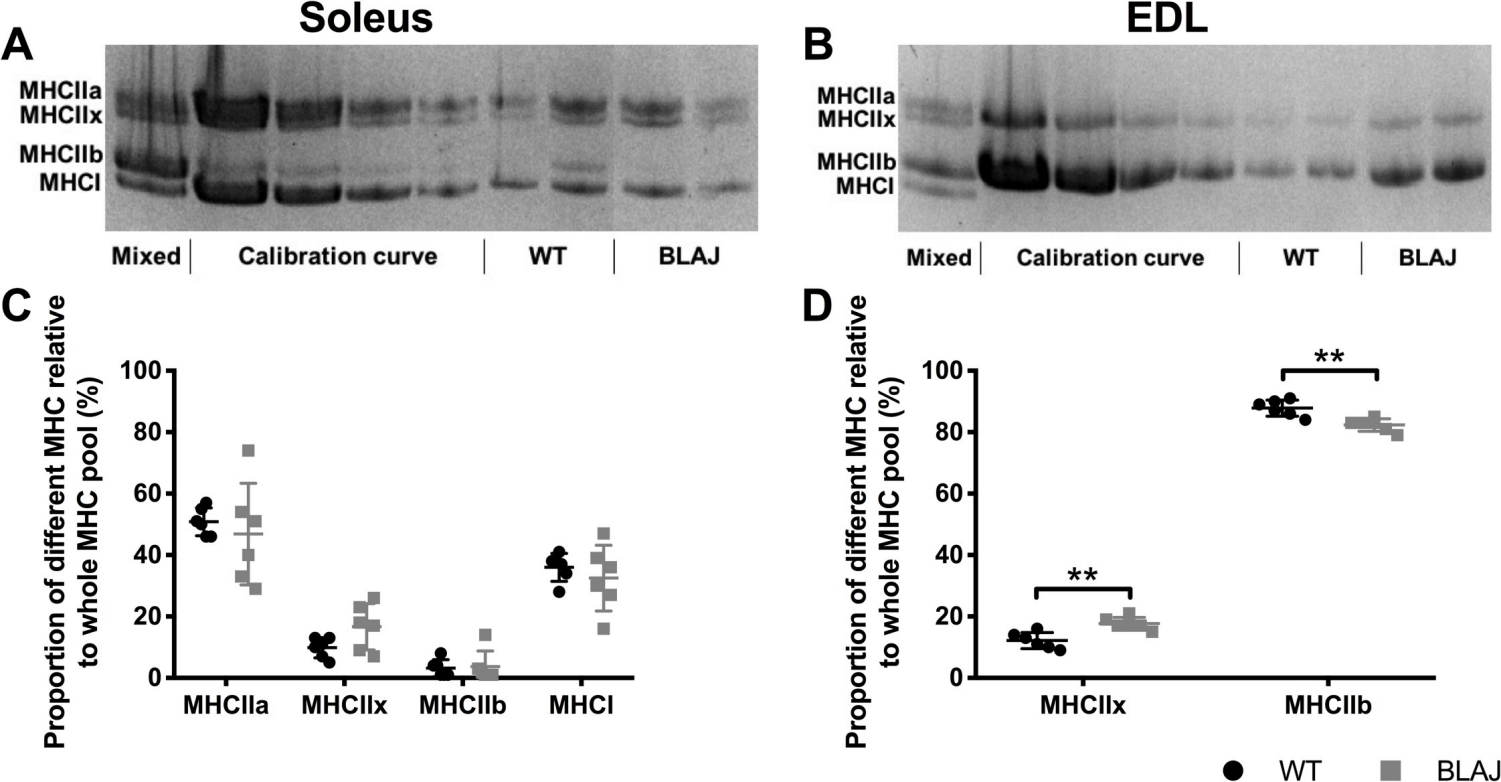
Myofibre myosin heavy chain (MHC) composition of soleus and EDL muscle from WT and BLAJ mice aged 10 months (*n* = 6). Representative MHC gels (A, B) for soleus and EDL muscles, loaded with a pooled sample (Mixed) used to generate the Calibration curve (see Methods), with (C, D) showing percentage of different MHC in soleus and EDL muscles. ** BLAJ significantly different to WT (*p*s < 0.01). Data are presented as individual values with horizontal lines indicating mean ± SD.

Analyses of the proportions of MHC isoforms in the soleus showed no significant differences in composition between the strains (*p*s > 0.05) (Fig 7). Dysferlin-deficiency significantly impacted the myofibre type composition of the EDL muscle, whereby the BLAJ EDL had a significantly larger proportion (+5.5%) of the type IIx MHC isoform and a smaller proportion (-5.5%) of the type IIb isoform (*p*s < 0.01), compared with WT.

## Discussion

The main findings of this study are that dysferlin-deficiency for male BLAJ mice aged 10 months (i) had a limited impact on our measures of *in vivo* muscle function, (ii) greatly increased the mass of BLAJ soleus muscles, and (iii) had myofibre type specific alterations of *ex vivo* muscle function for BLAJ soleus and EDL muscles. These findings are discussed in detail below.

### Limited impact of dysferlin-deficiency on in vivo measures of function in BLAJ mice

Normalised forelimb grip strength was unaffected in the 10 month old BLAJ (compared with WT) mice, a finding consistent with those in 9 month old A/J mice [26]. While increased forelimb grip strength (+63%) was reported in 2–6 month old dysf^-/-^ SJL/J mice [30], this difference may be due to the choice of C57Bl/6J mice as a control strain for the SJL/J mice (since dysferlin positive control SJL/J mice were not available at the time of their experiments).

Nonetheless, other studies have reported significantly lower grip strength in BLAJ mice (compared with WT) at ages older than 13 months [28, 29]. The absence of significant differences in our measures of BLAJ forelimb grip strength at 10 months of age may be due to the fact that forelimb muscles are less affected than hindlimb muscles in early stages of the disease [20] and greater disease progression may be required to show an effect on this parameter of *in vivo* muscle strength in older BLAJ mice.

Normalised grip strength is used to control for differences in body mass, under the assumption that most mice have a similar proportion of muscle mass, although in dysf^-/-^ mice, this relationship between body mass and muscle mass may not hold, especially in older dysf^-/-^ mice where fat replacement of myofibres is an increasing feature after about 8 months of age [5, 17]. Consequently, we examined the Pearson bivariate correlations between raw grip strength (average of 4 tests) and body mass for each group of mice (WT, BLAJ) and found no correlation. Since forelimb grip strength is dependent on many factors other than muscle strength, including behavioural and experimental variation [38], the usefulness and relevance of grip strength as a measure of muscle function in dysf^-/-^ mice should be carefully considered.

To further measure overall muscle function of BLAJ mice *in vivo*, we tested their capacity for voluntary low resistance wheel running over 2 weeks using mice sampled at 10 months of age; while running patterns were similar for both strains, average speed tended to be lower in the BLAJ, compared with WT (*p* = 0.055, partial η^2^ = 0.43, n = 5, 4 respectively). Our wheel running results are limited by the low sample sizes, due to limited availability of these old BLAJ mice, however these data were included since the trend of reduced speed in the BLAJ mice is suggestive of functional deficits and reflects similar findings of others. For example, swimming speeds of BLAJ mice were significantly slower than controls from 8 months of age [29]. Other studies have also shown significant reductions in voluntary wheel running distance [17] and open field behaviour [26, 27, 30] for dysf^-/-^ mice, aged 14, 2–6, 3–4 and 9 months respectively, indicative of functional impairments even in the mild pathology of dysf^-/-^ mice.

The function of individual BLAJ muscles with the most severe dystropathology, such as the limb girdle muscles psoas and quadriceps, is not easily assessed. Nevertheless, the quadriceps and psoas muscles are heavily involved in ambulation and thus open field behaviour and voluntary wheel running are useful measures of muscle function in BLAJ mice. It is important to consider the impact that many factors, including circadian rhythm and stress, can have on these measures [39], especially since mice are normally most active nocturnally and rest during the day (in contrast with most humans). Yet, the standard protocol of open field behaviour consists of placing a mouse in a test chamber for one hour during the morning hours for 4 successive days (see TREAT-NMD standard protocol “Behavioural and Locomotor Measurements Using Open Field Animal Activity Monitoring System–DMD_M.2.1.002”). This method does not take into account the important effects of circadian rhythm (on gene expression, metabolism and possible function and motivation) nor longer term activity of mice, and is stressful since it is normally performed during the daytime ‘rest period’. In contrast, the voluntary wheel running method keeps mice in the testing cage for the duration of the experiments, enabling continuous measurements of activity at night (when mice are naturally active) and over longer periods of time, with minimal handling of the mice (see TREAT-NMD standard protocol “Use of treadmill and wheel exercise for impact on mdx mice phenotype–DMD_M.2.1.001”). Thus, voluntary wheel running may be a more accurate measure of *in vivo* muscle function in mice, compared with classic open field behaviour tests (although behavioural testing during the nocturnal phase might be additionally informative).

### Increased mass of the BLAJ soleus

Dysferlin-deficiency had no impact on body mass of the 10 month old male mice, which is consistent with findings in A/J mice aged 9 months [26]. For muscle mass, the BLAJ soleus was significantly heavier than normal WT soleus. However, previous studies have reported variable results; no significant difference was reported in the mass of soleus muscles in WT and BLAJ mice aged 12 months [29], whereas soleus muscles from 2–6 month old SJL/J mice were reported to be significantly lighter compared to C57Bl/6J, though this finding may also be influenced by a strain difference [30]. It is of interest to consider the mechanisms that might contribute to the increased soleus mass in the old BLAJ mice.

From the perspective of histopathology, no conspicuous changes in histology were apparent for dysf^-/-^ soleus muscles aged 7 and 11 months, compared with WT muscles [33]. We also looked at the histology of a few frozen and fixed muscles for both strains (data not shown) and observed for the 10 months and older mice ‘apparently normal’ histology for all EDL and soleus muscles, with no marked differences between the BLAJ and WT muscles examined. However, a detailed formal analysis by light and electron microscopy is warranted to identify and quantify any (subtle) histopathological changes in the soleus muscle of older dysf^-/-^ mice.

The increased soleus mass in our old BLAJ mice may potentially be explained by oedema, with increased osmolarity arising from disturbed membrane dynamics and altered ion channel function or altered metabolite concentrations in slow myofibres, driving fluid accumulation within the muscle tissue. Oedema can also result from immune disturbances related to complement activation and deposition of the membrane attack complex (MAC) [40], and the immune response is complex in dysferlinopathies [41]. The MAC is a transmembrane pore that can alter membrane permeability resulting in swelling and lysis of the target cells. Dysf^-/-^ muscles of humans and mice have been shown to have disturbed complement activation, and surface deposition of complement and MAC (which is implicated in myofibre damage) even on ‘apparently undamaged muscles’ [32, 42, 43]. The MAC also drives atherosclerosis in apolipoprotein E knockout (ApoE null) mice on a high fat diet [44] and ApoE null mice crossed with dysferlin null mice show striking histopathology in many muscles with myofibres replaced by fat [33]: exacerbation of these histological features in double knock-out mice may reflect combined elevated levels of MAC in both null genotypes. Early studies reported high serum complement C3 levels of a different C3 allotype present in male SJL/J mice only, and altered degradation kinetics only in female SJL/J mice, compared with both genders of normal BALB/c mice and other inbred strains [45]. This impact of gender is important to consider for the complement system, since dysf^-/-^ female SJL/J mice were used in the study that reported downregulation of decay accelerating factor /CD55 [43], and fatty replacement of many muscles is more severe in female patients with dysferlinopathy [16].

Other MRI analyses in humans report muscle oedema (detected as hyper-intensity on STIR sequences) as an early consequence of dysferlinopathy; initially in the distal lower legs or in the proximal thigh muscles, followed by fatty degeneration [11]. It also seems pertinent that dysf^-/-^ skeletal muscle (compared with WT) is more susceptible to osmotic shock injury [46] with slower recovery to glycerol-induced osmotic shock [17]; such altered properties may also contribute to oedema in dysf^-/-^ muscles. The mechanisms and functional implications of the unusual increase in size of the slow soleus BLAJ muscle (comprised predominantly of type I and IIa myofibres) we observed warrants further investigation.

### Differential functional effects of dysferlin-deficiency on the soleus and EDL muscles

This study was particularly interested in the effect of dysferlin-deficiency on a wide range of measures of *ex vivo* muscle function. There were no differences in maximum tetanic force between WT and BLAJ soleus and EDL muscles, which is consistent with previous literature [25, 26, 32]. These findings indicate that higher stimulus frequencies do not produce observable differences in the force producing capacity of control and dysf^-/-^ muscle. If dysferlin-deficiency does disrupt normal Ca^2+^ handling [47], these effects were not evident at maximal stimulation frequencies, which is likely due to Ca^2+^ saturation of the myofilaments at these high frequencies [48]. Furthermore, the stimulus frequencies where maximum tetanic force is recorded do not reflect normal physiological activation [49]. We therefore also investigated muscle function across a range of stimulus frequencies.

Single twitch contractions showed significant differences between normal and dysf^-/-^ muscle function. Additionally, the results show novel differences in the impact of dysferlin-deficiency on slow and fast twitch muscle function. The BLAJ soleus had a significantly faster twitch contraction and relaxation, compared with WT muscle; whereas, the BLAJ EDL showed a slower relaxation and an increased maximum rate of force production. Similarly, both the BLAJ soleus and EDL had abnormal force-frequency relationships, whereby the mean force frequency curves were significantly shifted to the right (soleus) and left (EDL) compared to WT, particularly at the lower, more physiologically relevant stimulus frequencies. These force-frequency results reflect the differences found in TTP and 1/2RT of the BLAJ muscles. Compared with WT, the contraction times for the BLAJ soleus are shorter, so force summation would be reduced for a given stimulation frequency, whereas, the BLAJ EDL had a longer relaxation, likely contributing to higher force summation. Myofibre type specific differences were also shown in terms of delayed post fatigue recovery in the BLAJ soleus in the first 15 minutes. No significant effect of dysferlin-deficiency was observed in EDL fatigue or post fatigue recovery.

MHC myofibre typing showed that the differences in BLAJ soleus muscle contractions were not due to a change in myofibre type composition (as measured by MHC isoforms). As such, our results are indicative of disrupted EC coupling in the dysf^-/-^ soleus. This is consistent with past studies showing that dysferlin-deficiency is associated with ultrastructural changes to the T-tubules, and impairment of DHPR and RyR1 function in dysf^-/-^ skeletal muscle [6, 8, 9, 46, 50]. The variations in dysf^-/-^ soleus muscle function may be explained by myofibre type specific isoform variations in Ca^2+^ handling proteins, such as SERCA and calsequestrin, present in slow and fast twitch myofibres [51, 52].

In the BLAJ EDL, the relative increase in the proportions of MHC isoform ‘intermediate’ type IIx myofibres compared with ‘fast’ type IIb myofibres, may account for some of the differences in our *ex vivo* function measures, namely longer relaxation time and leftward shift of the force frequency curve. This shift in MHC isoform composition reflects findings in humans with advanced stage dysferlinopathy, where muscle biopsies from quadriceps, gastrocnemius, biceps, triceps, and deltoid muscles, displayed a type I myofibre predominance of up to 80%, suggesting a selective loss of type II myofibres [53]. In addition to an impact of dysferlin-deficiency on EC coupling that will affect muscle function, molecular disturbances that could preferentially impact the cellular and metabolic activities of slow or fast twitch myofibres need to be considered. In this context, studies in normal soleus and EDL muscles of adult male rats aging from 3 to 12 months, show substantial changes in many physiological and biochemical characteristics during early to mid-adulthood [37]; such post-growth changes in different muscles may affect the onset and progression of muscle diseases such as dysferlinopathy.

Previous experimental studies using dysf^-/-^ mice have focussed more on limb girdle muscles with pronounced histopathology, rather than distal limb muscles such as the soleus (without marked histopathology). However, we have shown that the soleus is in fact impacted by dysferlin-deficiency in terms of mass and contractile function. The soleus muscle from mice is reported to be similar to human skeletal muscle, due to the higher proportion of slow twitch myofibres [54], thus our murine results may have strong implications for understanding the dysferlinopathy pathology. Indeed, in the human disease, the soleus is one of the first muscles affected [55], with recent detailed MRI analyses of many muscles in human dysferlinopathy patients showing pathological changes in the soleus, due to adipocyte replacement, early in the disease progression [16]. The differences in severity of dystropathology progression in the soleus between the human disease and the mouse model may be due (in part) to the very different loading patterns present between large bipedal humans and very small quadrupedal mice, combined with impact of the far longer growth phase (~16–20 years depending on gender) and longevity of humans compared with mice.

Clearly further investigation into the nature of the impact of dysferlin-deficiency on the soleus and slow twitch myofibres in both species is of much interest. It would also be beneficial to investigate these functional characteristics of BLAJ skeletal muscle at other ages to better understand the progression of the disease. This new research focus on the nature of disease manifestation in different types of dysf^-/-^ myofibres, is likely to provide novel insights into the molecular basis for the disease that will influence the selection of specific muscles for design of future pre-clinical studies and, of much importance clinically may identify new drug targets for future therapies for dysferlinopathies.

## Supporting information

S1 File Whole dataset for this study. Each tab contains a particular dataset as follows: Body + muscle weights etc; In vivo muscle function; Ex vivo muscle function; Myofibre typing.
(XLSX)

S2 File Completed ARRIVE Guidelines Checklist. (PDF)
