# Correction: Dysferlin-deficiency has greater impact on function of slow muscles, compared with fast, in aged BLAJ mice

**DOI:** 10.1371/journal.pone.0286286

**Published:** 2023-05-22

**Authors:** Erin M. Lloyd, Hongyang Xu, Robyn M. Murphy, Miranda D. Grounds, Gavin J. Pinniger

After this article [1] was published, concerns were raised by the corresponding author that demarcation lines to indicate that lanes 7 and 8 in the myosin heavy chain SDS-PAGE gels in Fig 7A and 7B are noncontiguous were omitted. The corrected Fig 7 and legend are given below, where dashed lines indicate the noncontiguous lanes in Fig 7A and 7B. The original underlying gels for Fig 7A and 7B are provided here in S1 and S2 Files. The corresponding author stated that the lanes not included in Fig 7A and 7B include a treatment to animals that was not part of the study in article [1].

**Fig 7:**
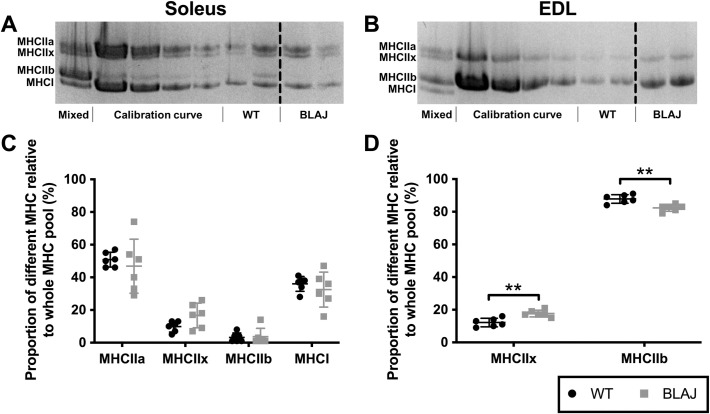
Myofibre myosin heavy chain (MHC) composition of soleus and EDL muscle from WT and BLAJ mice aged 10 months (*n* = 6). Representative MHC gels (A, B) for soleus and EDL muscles, loaded with a pooled sample (Mixed) used to generate the Calibration curve (see Methods), with (C, D) showing percentage of different MHC in soleus and EDL muscles. ** BLAJ significantly different to WT (*p*s < 0.01). Data are presented as individual values with horizontal lines indicating mean ± SD. Noncontiguous lanes from the same gel are separated by black dashed lines.

The authors apologize for the error(s) in the published article.

During follow-up, the authors informed the journal that corresponding author GJP is deceased. At the time of publication of this notice, the corresponding author for this article [1] is updated to Erin M. Lloyd, erin.lloyd@uwa.edu.au.

## Supporting information

S1 File Original underlying gel for Fig 7A where the lanes not included in Fig 7A are shown in blue boxes. (JPG)

S2 File Original underlying gel for Fig 7B where the lanes not included in Fig 7B are shown in blue boxes. (JPG)
