# Supplementary figures and images for: Correction: Dysferlin-deficiency has greater impact on function of slow muscles, compared with fast, in aged BLAJ mice
Source: PLoS One. 2023 May 22;18(5):e0286286. doi: 10.1371/journal.pone.0286286 (PMC10202262; doi:10.1371/journal.pone.0286286)

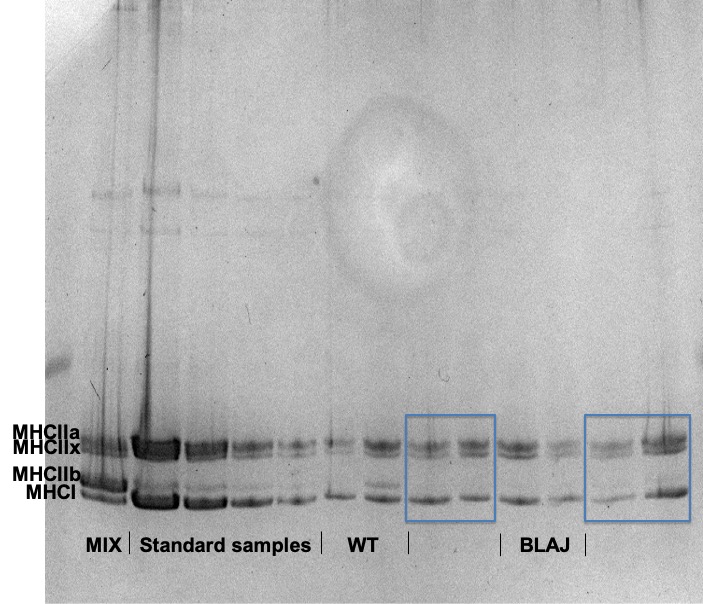

Supplement: S1 File — (JPG) [file pone.0286286.s001.jpg]

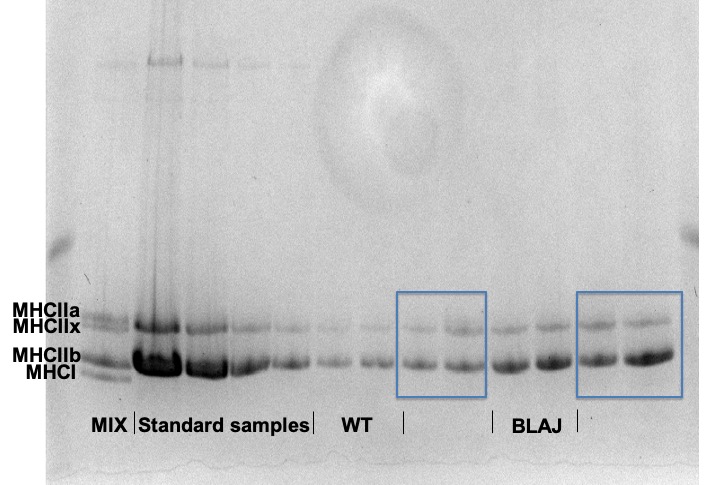

Supplement: S2 File — (JPG) [file pone.0286286.s002.jpg]
